# Letter to the editor. Re: “Coconut (Cocos nucifera) tree disease dataset: A dataset for disease detection and classification for machine learning applications,” Data in Brief, vol. 51, p. 109690, 2023

**DOI:** 10.1016/j.dib.2026.113154

**Published:** 2026-08-08

**Authors:** M. Sabrigiriraj, K. Manoharan

**Affiliations:** a Department of Information Technology, Hindusthan College of Engineering and Technology, Coimbatore, India; b Department of ECE, SNS College of Technology, Coimbatore, India

**Dear Editor,**

## 1 Overview

We read with interest the data article reporting a publicly available coconut tree disease image dataset for machine learning applications [1]. Public agricultural image datasets are important because they support reproducible research, comparative benchmarking, and broader participation in data-driven plant disease analysis. This is especially relevant in coconut disease research, where openly available datasets remain limited and are therefore likely to be reused in future studies.

During our attempt to use this dataset, we performed a dataset audit before model development to verify image integrity [2], assess sample uniqueness, and examine possible duplication-related concerns. Our audit identified 5798 readable images, and no corrupted or unreadable files were detected.

## 2 Details

We audited the published dataset by first checking image readability and then applying three duplicate-screening stages [3]. For strict exact duplicates, we computed both a SHA-256 file hash on the original file bytes and a SHA-256-pixel hash on the RGB pixel array; both methods produced the same result, namely 34 exact duplicate groups involving 68 images. Retaining one representative image per group removed 34 images and left 5764 images after exact duplicate removal. On the exact-cleaned set, we then applied perceptual hashing (pHash) and identified 139 perceptual-repeat groups involving 308 images; this stage was used for screening and overlap analysis rather than direct sample removal.

Finally, we screened for near-duplicates using deep visual features from an ImageNet-pretrained ResNet18 model. We compared images within each class using cosine similarity in FAISS and treated pairs with similarity ≥ 0.99 as near-duplicates. This identified 166 groups involving 463 images. After retaining one representative image per group, 297 images were removed and 5467 images remained as shown in Table 1. Representative detected pairs were also visually checked and were found to appear repeated or highly similar.

**Table 1:** Dataset audit summary.

**Audit step**	**Images removed**	**Images remaining**	**Notes**
Original readable dataset	—	5798	No corrupted or unreadable files detected
Strict exact duplicate removal	34	5764	34 groups involving 68 images; identical result under file-hash and pixel-hash matching
Perceptual repeat screening on exact-cleaned data	—	5764	139 pHash repeat groups involving 308 images; screening only, no direct removal at this stage
Near-duplicate removal after exact-cleaning	297	5467	166 deep feature-based near-duplicate groups involving 463 images; one representative retained per group

We recognize that some visually similar images may arise naturally from repeated observations during field data collection and may reflect normal intra-class variability under real agricultural conditions. Our audit documents these observations to support transparent dataset reuse and evaluation rather than to question the original data collection methodology.

**Fig. 1:**
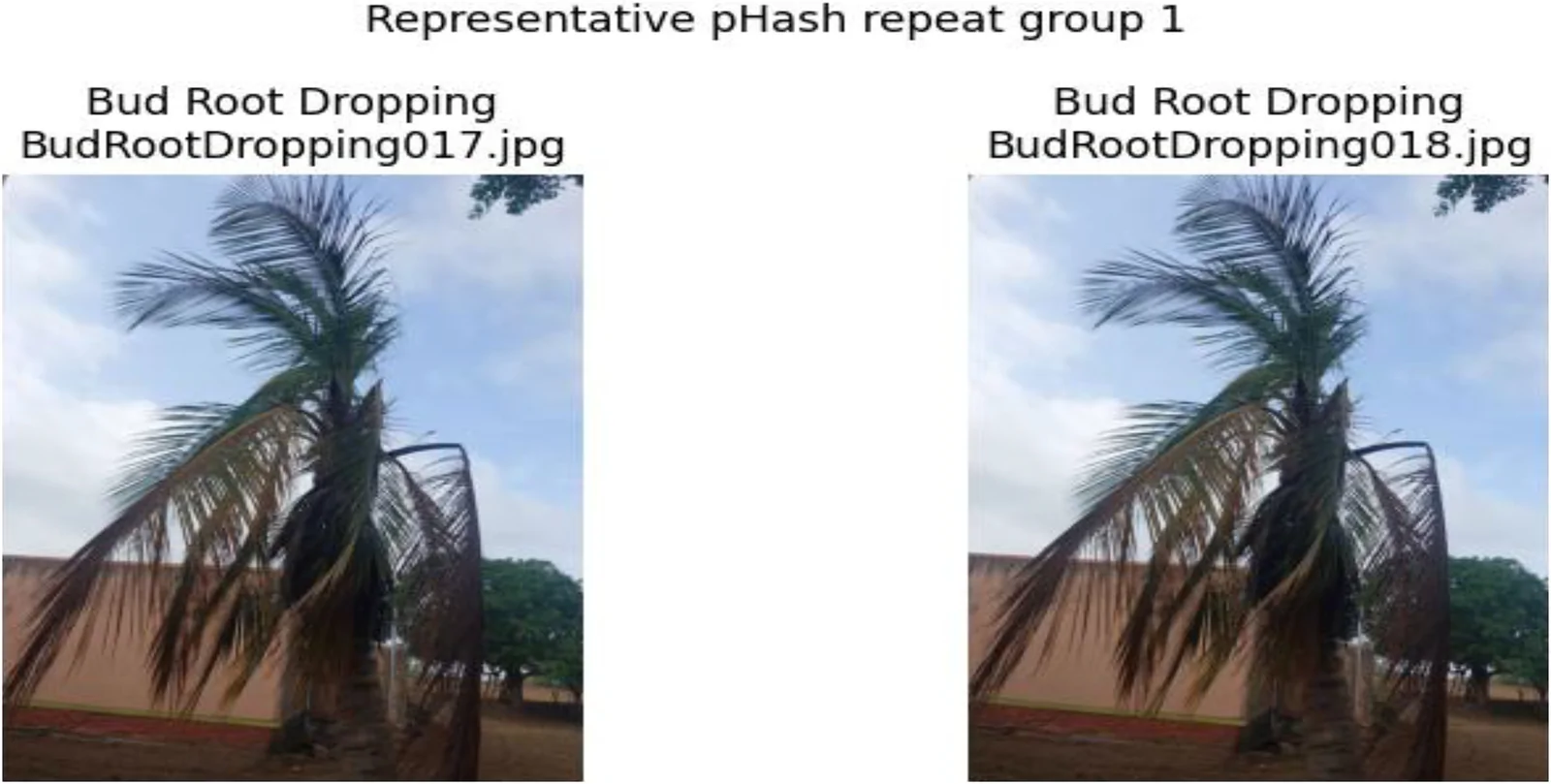
Representative example of a perceptually repeated image pair identified during dataset audit.

These findings indicate that the published dataset contains not only a small set of strict exact duplicates but also a larger number of perceptually repeated and highly similar samples. A sample is shown in Fig. 1. We also examined whether repeated content crossed class labels. In our audit, we did not identify cross-class perceptual repeats or cross-class high-similarity pairs, suggesting that the detected redundancy was primarily within classes.

We also observed that 50 perceptual-repeat groups crossed training and validation partitions under a random split, indicating that repeated visual content can cross evaluation boundaries when duplicate-aware controls are not applied. In our tested benchmark setting, the difference between conventional random splitting and leakage-aware group-based evaluation was small. Even when the observed performance difference is modest, documenting such repeated content remains important for duplicate-aware dataset reuse and reliable future benchmarking. Accordingly, our intention is not to claim a large distortion of previously reported results, but rather to document repeated content in a publicly reused dataset and to encourage duplicate-aware reuse practice. A class-wise summary of strict exact duplicate removal is provided in Supplementary Table 1.

## 3 Suggestions

The original dataset remains a useful public resource. The additional context provided by the original authors helps clarify the rationale behind the observed visual similarities and repeated observations within classes. Nevertheless, because the dataset is publicly distributed for reuse and benchmarking, reporting duplicate-aware audit findings may help future users understand the characteristics of the dataset and reduce the possibility of unintended evaluation leakage.

We therefore suggest duplicate inspection, duplicate-aware partitioning, and similarity-aware evaluation as practical precautions before model development and comparative benchmarking.

We stress that our intention is not to diminish the value of the dataset or the contribution of the original authors. Rather, because publicly available agricultural datasets are likely to be reused by many researchers, we felt it would be constructive to document these observations and provide additional context that may assist future users in selecting appropriate dataset partitioning and evaluation strategies. We believe that the original publication and the present audit together provide complementary information that supports transparent, reproducible, and informed reuse of the dataset.

## Ethics Statement

This letter is based on analysis of a publicly available dataset article and dataset files. It did not involve human participants, animals, or social media data.

## CRediT Author Statement

M. Sabrigiriraj: Conceptualization, Investigation, Formal analysis, Writing – original draft.

K. Manoharan: Investigation, Validation, Writing – review & editing.

## Declaration of Competing Interest

The authors declare that they have no known competing financial interests or personal relationships that could have appeared to influence the work reported in this letter.

## Appendix Supplementary materials

Image, application 1
