# Supplementary material for: Letter to the editor. Re: “Coconut (Cocos nucifera) tree disease dataset: A dataset for disease detection and classification for machine learning applications,” Data in Brief, vol. 51, p. 109690, 2023
Source: Data Brief. 2026 Aug 8;68:113154. doi: 10.1016/j.dib.2026.113154 (PMC13521133; doi:10.1016/j.dib.2026.113154)
Supplement: Supplementary file 1 [file mmc1.docx]

**Supplementary Table S1. Class-wise strict exact duplicate removal summary**

| Class | Readable images | Images removed after exact duplicate removal | Images after exact duplicate removal |
| --- | --- | --- | --- |
| Bud Root Dropping | 514 | 0 | 514 |
| Bud Rot | 470 | 0 | 470 |
| Gray Leaf Spot | 2135 | 17 | 2118 |
| Leaf Rot | 1673 | 16 | 1657 |
| Stem Bleeding | 1006 | 0 | 1006 |
| Total | 5798 | 34 | 5764 |
